# Kindlin-2 mediates mechanotransduction in bone by regulating expression of Sclerostin in osteocytes

**DOI:** 10.1038/s42003-021-01950-4

**Published:** 2021-03-25

**Authors:** Lei Qin, Xuekun Fu, Jing Ma, Manxia Lin, Peijun Zhang, Yishu Wang, Qinnan Yan, Chu Tao, Wen Liu, Bin Tang, Di Chen, Xiaochun Bai, Huiling Cao, Guozhi Xiao

**Affiliations:** 1 Department of Biochemistry, School of Medicine, Guangdong Provincial Key Laboratory of Cell Microenvironment and Disease Research, Shenzhen Key Laboratory of Cell Microenvironment, Southern University of Science and Technology, Shenzhen, China; 2 Department of Biomedical Engineering, Southern University of Science and Technology, Shenzhen, China; 3 Research Center for Human Tissues and Organs Degeneration, Shenzhen Institutes of Advanced Technology, Chinese Academy of Sciences, Shenzhen, China; 4 Department of Cell Biology, School of Basic Medical Sciences, Southern Medical University, Guangzhou, China

**Keywords:** Mechanisms of disease, Calcium and phosphate metabolic disorders

## Abstract

Osteocytes act as mechanosensors in bone; however, the underlying mechanism remains poorly understood. Here we report that deleting Kindlin-2 in osteocytes causes severe osteopenia and mechanical property defects in weight-bearing long bones, but not in non-weight-bearing calvariae. Kindlin-2 loss in osteocytes impairs skeletal responses to mechanical stimulation in long bones. Control and cKO mice display similar bone loss induced by unloading. However, unlike control mice, cKO mice fail to restore lost bone after reloading. Osteocyte Kindlin-2 deletion impairs focal adhesion (FA) formation, cytoskeleton organization and cell orientation in vitro and in bone. Fluid shear stress dose-dependently increases Kindlin-2 expression and decreases that of Sclerostin by downregulating Smad2/3 in osteocytes; this latter response is abolished by Kindlin-2 ablation. Kindlin-2-deficient osteocytes express abundant Sclerostin, contributing to bone loss in cKO mice. Collectively, we demonstrate an indispensable novel role of Kindlin-2 in maintaining skeletal responses to mechanical stimulation by inhibiting Sclerostin expression during osteocyte mechanotransduction.

## Abstract (web summary)

Qin et al. show the involvement of Kindlin-2 in osteocyte mechanotransduction. They show Kindlin-2 mediates skeletal responses to mechanical stimulation through Smad 2/3 mediated inhibition of Sclerostin.

## Introduction

Bone constantly remodels in response to mechanical forces during physical exercise and daily life. This concept is the well-known “Wolff’s law” introduced by German anatomist and surgeon Julius Wolff in the 19th century^1^. This golden rule for bone remodeling is now widely accepted to explain the force-induced bone formation process and disuse-induced bone loss in humans^2–4^ and animals^5^. The mechanical forces applied on bone tissue modify both bone structure and bone strength^6^. Even though the Wolff’s law for mechanical bone remodeling is well accepted and utilized in modern clinical practices^7,8^, mechanisms behind it are still poorly understood.

Osteocytes, as the major and long-lived cell type in bone environment, have been considered as the multifunctional regulators in skeletal tissue^9^, such as the origin of local calcium abundancy^10,11^ and the endocrine center for phosphate metabolism^12,13^. For the past two to three decades, osteocytes are gradually gaining more attentions from a “passive placeholder” to be the major orchestrator for bone mechanobiology^14–17^. Embedded in the mineralized extracellular matrix (ECM), osteocytes sense various environmental physical stimuli, such as direct bending, compression, osmotic pressure, and shear stress derived from fluid flow in the lacuno-canalicular system (LCS)^18^. During mechanical experiences, osteocytes take use of several molecular mechanosensors to transmit external forces into internal biochemical reactions. These mechanosensors include osteocyte cytoskeleton, dendritic processes, integrin-based focal adhesions (FA), connexin-based gap junctions, primary cilium, ion channels, et al.^17^.

Among all the osteocyte mechanosensors reported so far, FA is the key player in bone mechanobiology^19^. Consisting of more than 200 proteins^20^ and extensive protein–protein interactions in adhesome^21^, FA links the external microenvironment and internal cellular cytoskeleton^22,23^. Structurally and functionally, FA mediates bidirectional controls for cell mechanobiology^17,19,24^. On one hand, the “outside-in” signaling indicates that the external ECM and physical environment control integrin activity, FA-associated protein dynamics, cell cytoskeleton, and overall cellular responses to mechanical signals. On the other hand, the “inside-out” signaling states that the internal cellular status can also influence FA-associated protein abundancy and distribution, FA composition, and integrin sensitivity in ECM binding and degradation.

As one important FA protein, Kindlin-2 serves as a protein scaffold for multiple and dynamic protein–protein interactions in FA^25^. First, Kindlin-2 regulates the bidirectional integrin signals^26^ through direct protein–protein interaction with integrin β1 and integrin β3 subunits^27–30^. Second, Kindlin-2 also has direct interactions with other FA complex proteins, such as Migfilin^25^, Integrin-like kinases^31^, Talin, and Paxillin^32^. Third, Kindlin-2 is reported with direct binding to actin cytoskeleton^33,34^ and connects with multiple actin-associated proteins, such as Arp2/3^35^ and RhoGDIα^36^. As a consequence, Kindlin-2 is largely involved in mechano-related processes, such as FA formation, cell-ECM adhesion, cell spreading, and cell migration^27,29,32,37^. However, little is known so far for the function of Kindlin-2 in mechanobiology in vivo.

In mammals, Kindlin-2 is reported with ubiquitous expression in major tissues^38,39^. Global deletion of *Kindlin-2* gene caused embryonic lethality at peri-implantation stage in mice at E7.5^26^. Kindlin-2 is reported with indispensable roles at different tissue backgrounds, such as mesenchymal stem cell (MSC) fate decision^40,41^, muscle development^42–45^, pancreatic development^46^, adipogenesis and lipid metabolism^47^, podocyte structure and function in kidney^36^. In bone tissue, Kindlin-2 regulates chondrogenesis^41^, osteogenesis and osteocyte survival^48^. Our group previously reported that osteocyte-specific *Kindlin-2* deletion through 10-kb mouse dentin matrix protein 1 (*Dmp1-Cre)* caused obvious osteopenia in mice, which deteriorates bone accrual and homeostasis and parathyroid hormone bone anabolism^48,49^. Deletion of *Kindlin-2* in osteocytes is tightly associated with increased osteocyte apoptosis and abnormal expression of secretary Sclerostin protein in these cells^48^. Sclerostin is a well-known negative regulator for bone formation in mechanical stimulation^50^. The expression of Sclerostin in osteocytes and its secretion in blood circulation have been widely used as indicators for osteoporosis and fracture risks in both animals^51,52^ and humans^53–57^. However, detailed mechanisms behind osteocyte Kindlin-2 and Sclerostin in the regulation of bone homeostasis are still unclear.

The aim of this study was to determine whether Kindlin-2 in osteocytes plays an important role in mediation of bone mechanotransduction. Using in vitro and in vivo model systems, we demonstrate that osteocyte Kindlin-2 largely modulates bone responses to mechanical loading through downregulation of Sclerostin expression in osteocytes.

## Results

### Kindlin-2 deletion in osteocytes results in notable bone loss in weight-bearing long bones, but not in non-weight-bearing calvariae

**Fig. 1:**
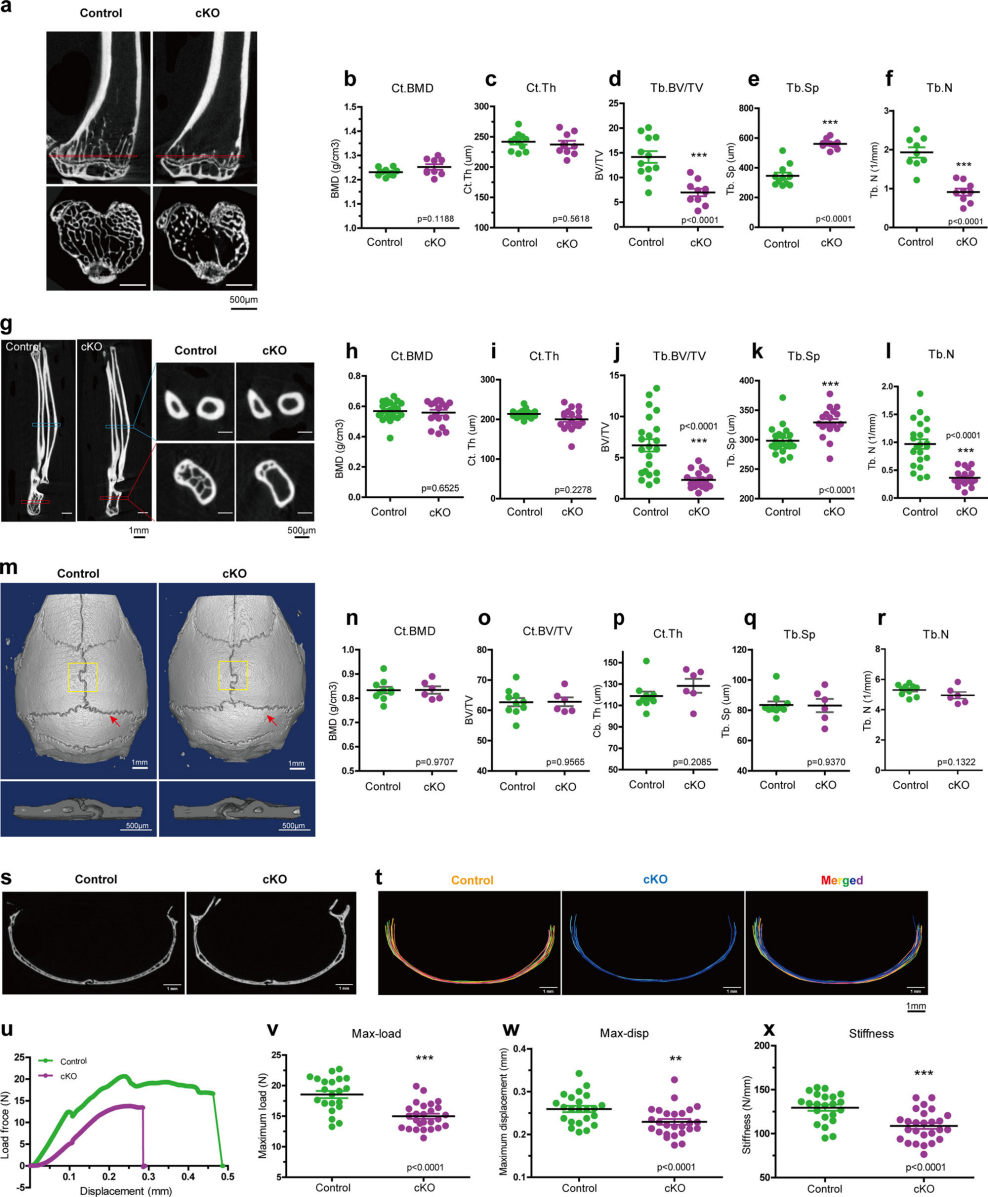
Kindlin-2 deletion in osteocytes results in remarkable bone loss in weight-bearing long bones but not in non-weight-bearing calvariae. **a** Micro-computerized tomography (μCT) images of the sagittal section and transverse section of proximal tibial bone from control (*Kindlin-2*^*fl/fl*^) and cKO (*Dmp1-Cre; Kindlin-2*^*fl/fl*^) mice. Red-squares indicate the region of interest for transverse sections in the sagittal images. **b**–**f** Quantification of μCT results of tibial cortical and trabecular bone in control and cKO mice. *N* = 10–12 for each group. **g** Representative μCT images for ulna cortical bone and trabecular bone of control and cKO mice. **h**–**l** Quantification of μCT data of ulna cortical and trabecular bone in control and cKO mice. *N* = 18–22 for each group. **m** Three-dimensional (3D) μCT of the whole calvaria from control and cKO mice (top figures). The ROI for μCT analysis is a 2 × 2 mm^2^ area in the center of the parietal bone (bottom figures), which is highlighted in yellow square. **n**–**r** Quantification of μCT data of calvaria ROI in control and cKO mice. *N* = 6–10 for each group. **s** μCT cross-section view of the suture of pariental and interpariental bones, as presented in **m** with red arrows. **t** Super-imposed cross-section image results for 10 control mice and 6 cKO mice. **u** Representative force-displacement curve for femurs from control and cKO mice. **v**–**x** Quantification data of max-load, max-displacement, and stiffness for the femurs from control and cKO mice. *N* = 22 for control mice; *N* = 26 for cKO mice. Bone samples were collected from 4-month-old male mice. *P < 0.05, **P < 0.01, ***P < 0.001, versus controls, Student’s *t* test. Results are expressed as mean ±  standard deviation (s.d.).

We recently generated osteocyte conditional *Kindlin-2* knockout mice (hereafter referred to as *Kindlin-2*^*Dmp1*^ or cKO) by breading the *Kindlin-2*^*fl/fl*^ mice with the *Dmp1*-Cre transgenic mice^48^. Our previous study demonstrated a striking osteopenia of *Kindlin-2*^*Dmp1*^ mice in distal femurs and lumbar spine (L4)^48^. We wondered whether this bone loss is universal for other parts of skeleton in cKO mice. We therefore performed micro-computerized tomography (μCT) analyses of other bones, including hindlimb tibia, forelimb ulna, and calvaria for cKO mice and their control littermates at 4 month age (Fig. 1), when their skeletal system is fully matured and the expression of *Dmp1-Cre* reaches to a relative high level^58^.

We first compared the bone mass from the cortical and distal trabecular bones of hindlimb tibia and forelimb ulna in control and cKO mice (Fig. 1a–l). Similar to our previous observations^48^, μCT analysis exhibited a remarkable bone loss in the trabecular bone of both tibia and ulna in cKO mice (Fig. 1a, g). Compared with their age-matched controls, cKO mice showed a 50.7% reduction in bone volume fraction (BV/TV), 62.2% increase in trabecular separation (Tb. Sp), and 52.9% reduction in trabecular number (Tb. N) in the trabecular bone of tibia (Fig. 1d–f). Moreover, ulna bone showed a 64.4% reduction in BV/TV, 10.4% increase in Tb.Sp, and a 62.5% reduction in Tb. N in the trabecular bone of cKO mice (Fig. 1j–l). Consistent with our previous observations in femurs^48^, there were no marked alterations in cortical bone mineral density (BMD) and cortical thickness (Ct.Th) in either tibia or ulna of the two genotypes (Fig. 1b, c, h, i). Detailed parameter detection from μCT analysis was listed in Supplementary Fig. 1b, c.

Next, we examined the calvaria difference between control and cKO mice. As presented in Fig. 1m–r, calvaria from 4-month-old male mice showed a comparable bone mass for all parameters between cKO and control mice, including BMD, BV/TV, Ct.Th, Tb.N, Tb.Sp, et al. (Supplementary Fig. 1a). To examine any possible structure differences in calvaria, we compared the μCT cross sections at the interphase between pariental and interpariental bones in control and cKO mice (Fig. 1s). Super-imposed images from control (in red) and cKO (in blue) revealed that the cross-section structure of calvariae was highly similar between the two genotypic mice (Fig. 1t).

Results from immunofluorescence staining showed that osteocytes from both femurs and calvariae of control mice expressed high level of Kindlin-2 protein, which was dramatically reduced in both bone types in age- and sex-matched cKO mice (Supplementary Fig. 2a, b). Likewise, western blotting using extracts from femurs and calvariae revealed high expression level of Kindlin-2 protein in control mice, which was significantly decreased in femurs and calvariae in cKO mice (Supplementary Fig. 2c). These results demonstrate that osteocytes of long bones and calvariae express high level of Kindlin-2 and that the *Dmp1-Cre* displays a similar Kindlin-2 deletion efficiency in osteocytes of both types of bones. Thus, it is unlikely that the lack of osteopenia in the calvariae of cKO mice is due to differences of Kindlin-2 expression and/or Dmp1-Cre activity in osteocytes between the two types of bones. Furthermore, we analyzed the bone mass of alveolar bone, which is derived from intramembrane ossification, like calvaria, but experiences frequent mechanical loading^59^. An obvious alveolar loss was observed in cKO mice, compared to control mice (Supplementary Fig. 3a–f). Together, these results suggest that the remarkable bone loss due to osteocyte Kindlin-2 deletion is restricted in weight-bearing bones, i.e., femur, tibia, ulna, and alveolar bone, but not in non-weight-bearing calvaria.

### Kindlin-2 loss in osteocytes impairs mechanical properties in mouse long bones

Wondering whether Kindlin-2 loss affects the long bone quality, we next tested the mechanical properties of femurs from 4-month-old control and cKO male mice through three-point-bending test. As presented in the load-displacement curve (Fig. 1u), femurs from control mice had higher maximum load (max-load, Fig. 1v) and higher maximum displacement (max-disp, Fig. 1w) than cKO mice. In this test, the average stiffness of femurs from control mice is 129.5 ± 3.569 N/mm (Fig. 1x). However, this number was decreased to 108.7 ± 3.373 N/mm in cKO femurs (Fig. 1x), which was a 16.62% reduction from control mice. Collectively, these results clearly demonstrate that Kindlin-2 loss impairs the mechanical properties in long bones. Femur, tibia, ulna, and lumber spine are the important load-bearing bones in body, which experience higher strain during daily physical activities than the flat calvaria^60,61^. Therefore, we hypothesized that the specific bone loss in cKO is associated with the mechanical environment in long bones and that osteocyte Kindlin-2 is crucial for the bone responses for mechanical stimulation.

### Kindlin-2 loss in osteocytes impairs responses of long bones to mechanical loading stimulation in mice

**Fig. 2:**
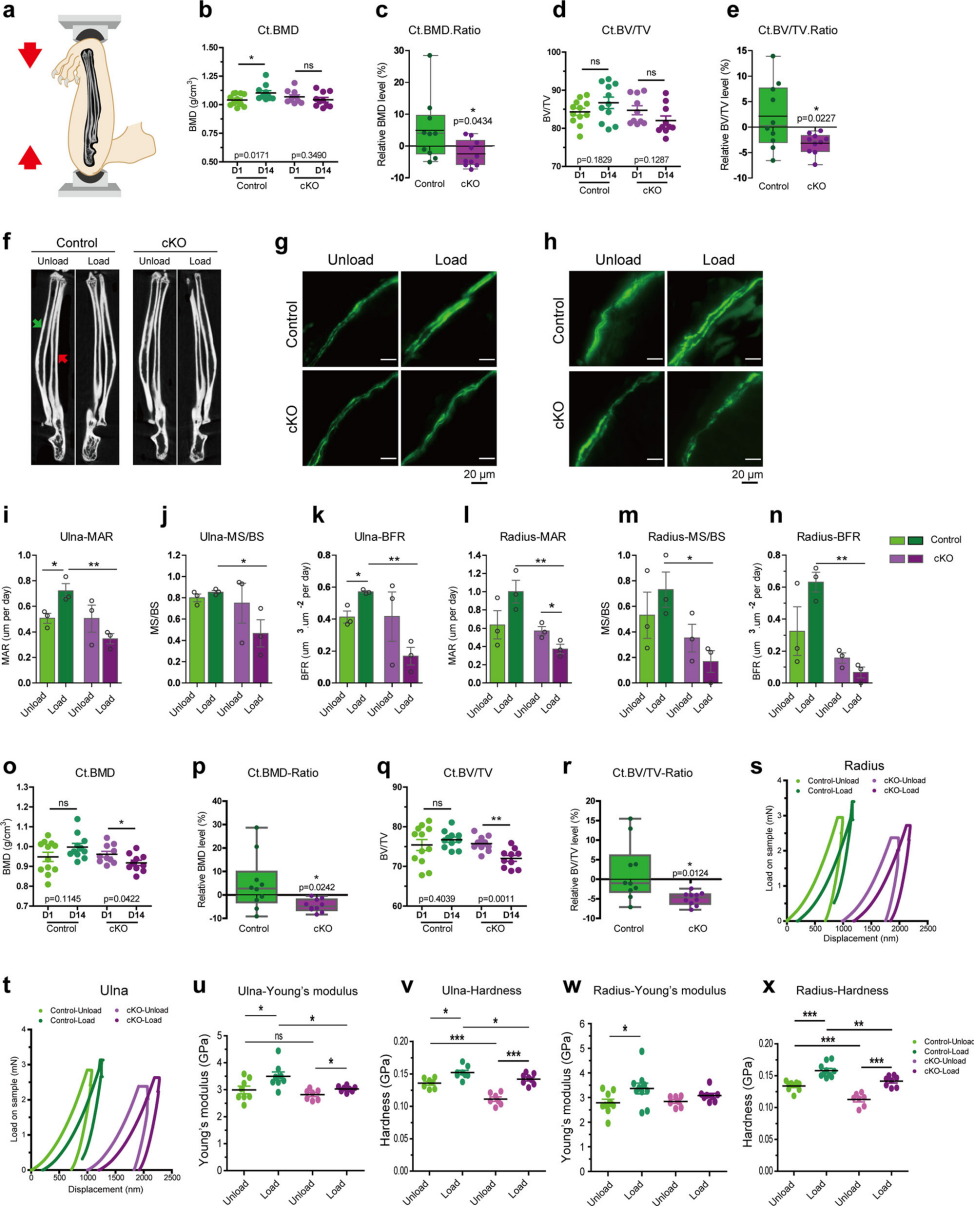
Kindlin-2 loss in osteocytes impairs skeletal responses to external mechanical ulna loading. **a** Illustration for ulna loading model. **b**–**e** Quantification of right ulna cortical bone before (D1) and after (D14) mechanical loading stimulation in control and cKO mice. *N* = 10–12 for each group. **f** μCT images for ulna and radius of unload (left) and load (right) limbs in control and cKO mice. Green arrow pointed to radius; red arrow pointed to ulna. In vivo double calcein labeling for bone formation detection in unload and load ulna (**g**), and in unload and load radius (**h**) of experimental mice. Statistical analysis for MAR, MS/BS, and BFR in unload and load ulna (**i**–**k**), and in unload and load radius (**l**–**n**) of control and cKO mice. *N* = 3 for each group. **o**–**r** Quantification analysis for right radius cortical bone before (D1) and after (D14) mechanical loading stimulation in control and cKO mice. *N* = 10~12 for each group. Representative force-displacement curves for nano-indentation over control and cKO ulna (**t**) and radius (**s**) samples. Young’s modulus and hardness of ulna (**u**, **v**) and radius (**w**, **x**) for control and cKO mice. *N* = 7–10 for each group. *P < 0.05, **P < 0.01, ***P < 0.001, versus controls, Student’s *t* test. Results are expressed as mean ± standard deviation (s.d.).

To test our hypothesis that specific loss in cKO long bones is associated with mechanical loading, we challenged cKO mice and their control littermates with two in vivo loading models. The first model we used was the ulna loading mouse model (Fig. 2a). In general, we loaded the right ulna of 4-month-old male mice with 6 times of 2.5 N cyclic mechanical compression within 2 weeks. Meanwhile, we traced the bone mass changes through in vivo μCT detection before (Day 1) and after (Day 14) loading, and monitored the bone formation with the double calcein labeling during loading experiments. In this model, the left ulnas were served as internal unload controls. As expected, we found that the BMD was increased in the load ulna of control mice after 2 week experiments (Fig. 2b). Surprisingly, mechanical loading failed to stimulate bone mass increment in cKO ulna. In the comparison of the percentage changes of bone mass after 14 days’ experiment, the cortical ulna of control mice displayed a 5% increase in BMD (Figs. 2c) and 2.1% increase in BV/TV (Fig. 2e), which are consistent with previously published results in wild-type animals^51,52^. However, the cortical ulna of cKO mice showed 2.4% decrease in BMD (Fig. 2c) and 3.2% decrease in BV/TV (Fig. 2e). In this model, in vivo ulna loading involves two parallel bones in mouse forelimb (Fig. 2f), i.e., ulna (red arrow in Fig. 2f) and radius (green arrow in Fig. 2f). Since radius also experiences mechanical loading and shares ~23–35% of force during external mechanical stimulation^62^, we also examined the bone density changes of load radius in both control and cKO mice (Fig. 2o–r). From μCT analysis, radius from control mice showed slight increases in BMD and BV/TV in the cortical bone (Fig. 2o, q). Similar to ulna results, cKO mice showed significant bone loss in radius by −4.53% and −4.93% of BMD and BV/TV changes, respectively (Fig. 2p, r). Full set of μCT scanning results for loading samples were summarized in Supplementary Fig. 5 (Supplementary Fig. 5a, b). Together, these results demonstrate that mechanical ulna loading enhances ulna and radius bone formation in control mice, but induces bone loss in cKO mice.

To further confirm the force-induced bone loss in ulna and radius, we performed the double calcein labeling experiments to measure the in vivo bone-formation activities in load ulnas and unload ulnas after mechanical stimulation. As shown in Fig. 2g, h, mechanical loading largely increased the calcein labeling intensity, mineral apposition rate (MAR), and bone formation rate (BFR) in both ulna and radius of control mice ulna. Whereas, cKO mice had reduced MAR, BFR, and mineralizing surface per bone surface (MS/BS) for the load ulna (Fig. 2i–k) and load radius (Fig. 2l–n). Notably, even though the unload ulna did not show statistically significant differences between control and cKO mice, external mechanical loading lowered the bone formation rate in cKO load ulna and radius compared to that in the control load ulna.

We next wanted to know whether the mechanical properties of forelimbs were affected after loading in the presence and absence of osteocyte Kindlin-2. To this end, we conducted nano-indentation experiments over non-decalcified unload and load bone samples from control and cKO mice and tested the Young’s modulus and hardness of cortical bone of ulna (Fig. 2t–v) and radius (Fig. 2s, w, x). Results from nano-indentation showed that both ulna and radius from cKO mice had significantly lower hardness compared to that in their control littermates, but no obvious Young’s modulus difference was observed between these two genotypes. After mechanical loading, the Young’s modulus of ulna and radius from control mice was increased 16.9% (Fig. 2u) and 20.8% (Fig. 2w), respectively. Comparably, the Young’s modulus of cKO mice had only 7.5% increase (Fig. 2u) for ulna and no significant change for radius (Fig. 2w). Moreover, the Young’s modulus and Hardness of load ulna and load radius between control and cKO mice showed significant difference, which indicates that mechanical loading enlarges the difference of bone mechanical properties in control and cKO mice. Therefore, these data reported so far suggest that deletion of Kindlin-2 in osteocytes affects not only bone mass, but also bone mechanical property enhancement during force-adaptation process in mouse forelimbs.

To further determine the involvement of osteocyte Kindlin-2 in the mediation of bone mechanotransduction, we performed the second in vivo tibia loading model with control and cKO mice (Supplementary Fig. 4). We loaded cyclic compression (4 Hz, triangular waveform) with 9.0 N peak force on the right tibia of 4-month-old male mice for 2 weeks. Consistent with ulna loading results, control mice showed obvious bone mass increase, whereas cKO mice exhibited a clear reduction in BV/TV after mechanical loading (Supplementary Fig. 4a–d). Furthermore, the double calcein labeling experiments demonstrated significantly reductions of MAR and BFR in the load tibia of cKO mice, compared to that of control mice (Supplementary Fig. 4e–g). Detailed parameter detection from μCT analysis in loading experiments were summarized in Supplementary Fig. 5.

Collectively, both ulna and tibia loading results demonstrate that mice with osteocytes-specific Kindlin-2 deletion fail to properly respond to mechanical stimulation. These results suggest a critical role for Kindlin-2 in osteocyte in bone formation triggered by mechanical stimulation.

### Control and cKO mice display similar bone loss in response to mechanical unloading; but cKO mice fail to restore lost bone after reloading

**Fig. 3:**
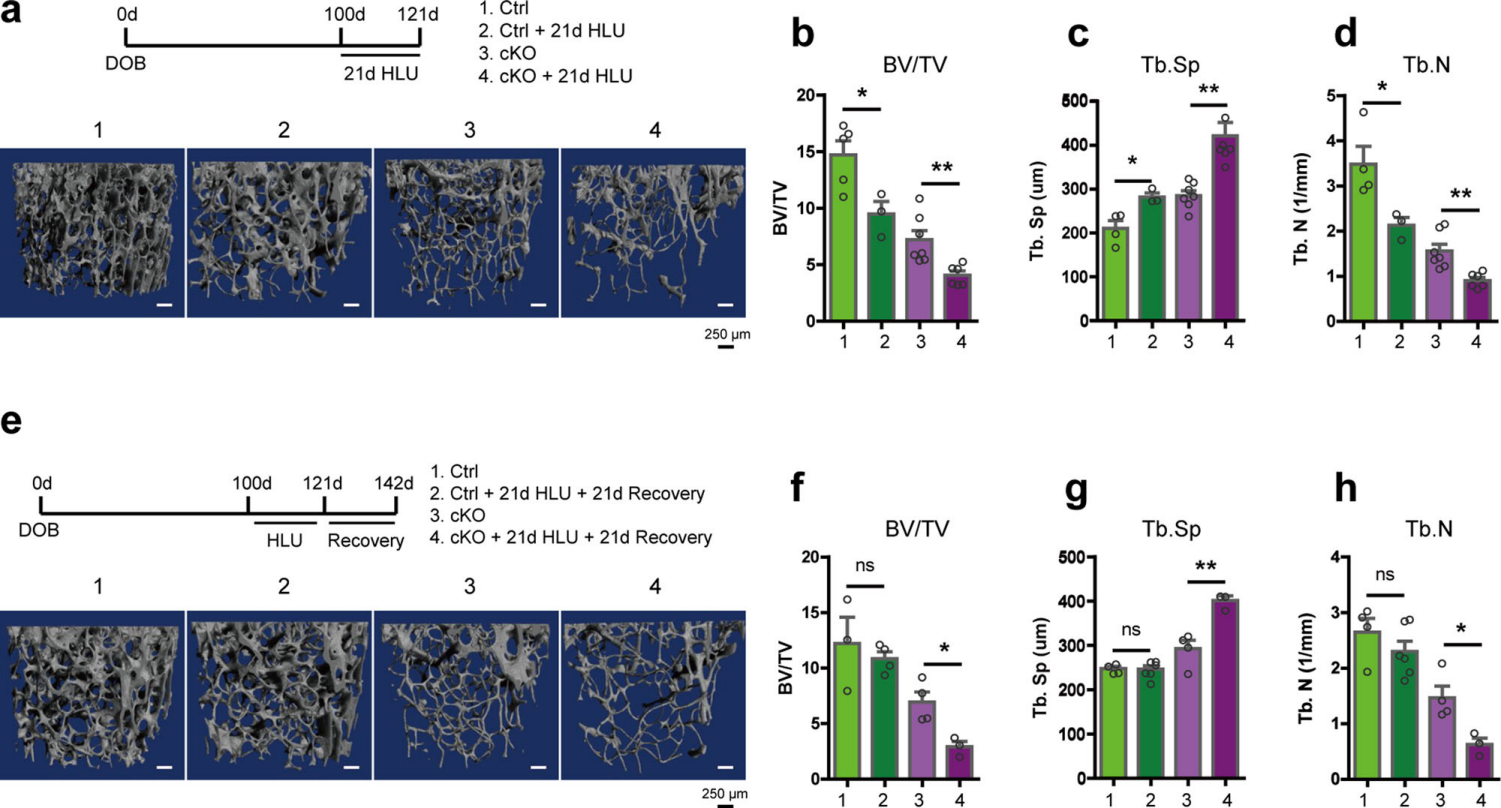
Mice with Kindlin-2 loss in osteocytes fail to restore the hindlimb unloading (HLU)-induced bone loss. **a** μCT 3D reconstruction of distal femur trabecular bone from control and cKO mice before and after 21 days’ HLU experiment. *N* = 3–7 for each group. **b**–**d** Quantification of the trabecular bone from control and cKO mice in HLU experiments. **e** μCT 3D reconstruction of distal femur trabecular bone from control and cKO mice in 21 days’ HLU and 21 days’ recovery experiment. **f**–**h** Quantification of the trabecular bone from control and cKO mice in HLU-recovery experiments. *N* = 3–6 for each group. *P < 0.05, **P < 0.01, ***P < 0.001, versus controls, Student’s *t* test. Results are expressed as mean ± standard deviation (s.d.).

After confirming that the skeleton of cKO mice fails to properly respond to mechanical loading, we wondered whether Kindlin-2 loss affects osteocyte sensation to unloading conditions. To do so, we conducted 21 days hindlimb-unloading (HLU) experiments with 14-week-old male control and cKO mice and detected bone mass changes in the hindlimb femurs (Fig. 3a). As shown in Fig. 3a, both control and cKO mice exhibited significant bone mass alternations in their femurs with substantial reductions of BV/TV and Tb.N, and marked increase of Tb. Sp after HLU experiments. Quantitatively, the percentage changes for bone mass loss after HLU were ranged from 33.8 to 36.1% (−35.6% for BV/TV, −36.1% for Tb.N, and +33.8% for Tb.Sp) in control mice, and 41.8–47.8% (−43.6% for BV/TV, −41.8% for Tb.N, and +47.8% for Tb.Sp) in cKO mice (Fig. 3b–d). The bone loss appeared to be slightly larger in cKO mice than that in control mice, but loss-of-Kindlin-2 seems to  have no effects on unloading sensation and disuse-induced bone loss.

To further investigate whether Kindlin-2 is involved in mechanical reloading sensation, we extended the HLU experiments with extra 21 days recovery, which allows the experimental animals to freely explore and experience mechanical loading in daily activities (Fig. 3e). As expected, the control mice restored their bone mass with comparable BV/TV, Tb.Sp, and Tb.N in control and HLU-recovery group (Fig. 3f–h). However, the cKO mice failed to regain their bone mass at the recovery stage, but still presented significant decreases in BV/TV (−57.9%) and Tb.N (−57.5%), and increase in Tb. Sp (37.1%) in HLU-recovery group (Fig. 3f–h). These results further demonstrate that compared to unloading sensation, Kindlin-2 is more responsible for mechanical loading sensation in osteocytes.

### Kindlin-2 deletion reduces osteocyte dendrite formation and cell attachment in vitro and in bone

**Fig. 4:**
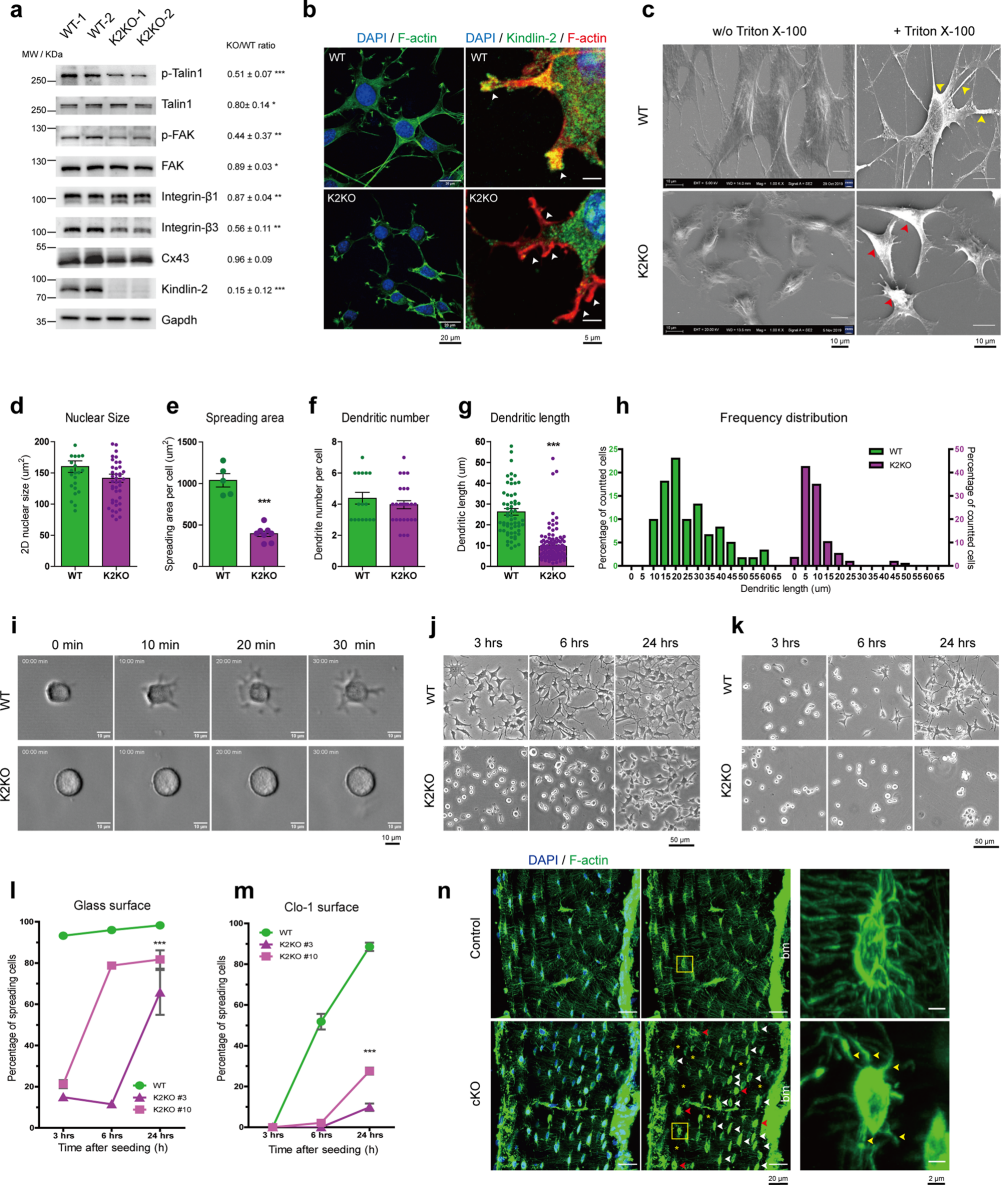
MLO-Y4 cells with Kindlin-2 deletion exhibit dramatic focal adhesion defects and cytoskeleton alternation. **a** Western blot results for wild-type (WT) and Kindlin-2 knockout (K2KO) MLO-Y4 cells. **b** Immunofluorescence (IF) staining for DAPI, F-actin, and Kindlin-2 in WT and K2KO cells. **c** Scanning electron microscopy (SEM) images for the cytoskeleton of WT and K2KO cells with and without TX-100 treatment. **d**–**h** Quantitative analysis for nuclear area, spreading area, dendritic number, dendritic length, and the frequency distribution of dendritic length of WT and K2KO MLO-Y4 cells. **i** Representative images from live tracking of single cell spreading within the first 30 minutes after seeding of WT and K2KO MLO-Y4 cells on uncoated glass bottom dishes. Representative images of WT and K2KO spreading and attachment on non-coated glass surface (**j**) and Col-1 coated surface (**k**) at 3, 6, and 24 h after seeding. Statistical analysis of percentage of spreading cells for WT and K2KO cells on glass surface (**l**) and Col-1 coated surface (**m**) at 3, 6 and 24 h after seeding. **n** Rhodamine-Phalloidin staining for F-actin over 20 μm tibia bone sections from Control and cKO mice. Quantitative results from at least three independent experiments. *P < 0.05, **P < 0.01, ***P < 0.001, versus controls, Student’s *t* test. Results are expressed as mean ± standard deviation (s.d.).

To understand the mechanism behind Kindlin-2 in the regulation of osteocyte mechanical responses, we next utilized a widely used osteocyte-like cell line, MLO-Y4 cells^63^, as an in vitro model for further studies. We previously deleted *Kindlin-2* gene in MLO-Y4 cells through the Crispr-Cas9 technology^48^. Considering the importance of Kindlin-2 in FA complex^26,28^ and the participation of FA in osteocyte mechanobiology^17^, we first tested the changes of FA components under different Kindlin-2 genetic backgrounds. Through western blotting, we found that, compared to the wild-type (WT) cells, *Kindlin-2* knockout (K2KO) led to remarkable reductions in the expression of integrin and FA-associated proteins in osteocytes (Fig. 4a). The expression of two integrin isoforms that were highly detected in osteocytes, i.e. integrin β1 and integrin β3^23^ and several Kindlin-2 binding proteins, i.e. Talin1^29,32^, focal adhesion kinase (FAK)^64^ and their phosphorylated isoforms were decreased by the Kindlin-2 ablation in MLO-Y4 cells. Interestingly, the expression of Connexin-43, an important mechanosensor in osteocyte biology^17^, was not markedly altered in K2KO MLO-Y4 cells.

With this broad influence in FA and FA-associated proteins, Kindlin-2 deletion in osteocytes affected the focal adhesion formation and cellular morphology in MLO-Y4 cells (Fig. 4b, c). As shown in Fig. 4b, Kindlin-2 loss caused dramatic alternation in FA, whose loosen and less-concentrated attachment to substrate (white arrows) indicated a reduced tension in K2KO FA sites. Moreover, Kindlin-2 deletion also brought notably morphological changes in MLO-Y4 cells with dramatic reductions in spreading area (Fig. 4e) and dendritic length (Fig. 4g). Specifically, WT MLO-Y4 cells had a broader frequency distribution for single dendritic length than K2KO cells (Fig. 4h). The majority of dendritic length in WT cells ranged from 15 to 20 μm, whereas K2KO cells only extended dendrites in 5–10 μm length (Fig. 4h). Furthermore, we found that Kindlin-2 controls plasma membrane continuances and cytoskeleton arrangement in MLO-Y4 cells. As shown in the scanning electron microscopy (SEM) images in Fig. 4c, WT MLO-Y4 cells had large spreading area with normal and continuous plasma membrane, while K2KO cells displayed smaller spreading area with clear membrane curvature under SEM. Because the presence of nonionic detergent Triton X-100 (TX-100) can induce cell membrane destabilization, permeabilization, and lysis^65^, it has been used for plasma membrane removal and in situ cytoskeleton detection^66^. To observe the cellular cytoskeleton, we pre-incubated WT and K2KO cells with TX-100 before fixation and SEM imaging. We found that WT cells presented less and sparse cytosol components around nucleus, but high and dense cytoskeleton component at the dendrites (yellow arrows). However, in K2KO cells, this difference of component distribution disappeared and the whole cells were filled with dense cytoskeleton (red arrows).

Based on these cytoskeleton and FA defects in K2KO cells, we further tested the spreading and attachment ability for MLO-Y4 cells at different genetic backgrounds. We first traced the cell spreading dynamics within the first 30 min after cell seeding (Fig. 4i and Supplementary movie 1). Through live cell imaging, we found that, right after cell seeding, WT cells rapidly protruded outside and formed multiple dendrites within the first 30 min. However, K2KO cells failed to generate any protrusions or form any dendrites within the first 30 min (Fig. 4j, k). We next monitored the spreading and attachment of MLO-Y4 cells at different time points. As presented in Fig. 4j, on uncoated glass surface, WT cells achieved 93.3% of attachment 3 h after seeding and reached to 98.3% 24 h after seeding. However, K2KO cells only reached to 18.1% of attachment at 3 h, with increased percentage at 6 h and reached to 73.7% 24 h after seeding (Fig. 4l). Since Collagen-I (Col-I) is one abundant ECM protein^67^ that activates integrin pathways^68^, we also tested the attachment ability of MLO-Y4 cells on Col-I coated surface. Interestingly, this spreading difference was enlarged on Col-1 coated surface. As shown in Fig. 4k, m, WT cells needed some time to adapt to Col-1 coated surface and finally reached up to 88.5% 24 h after seeding. However, K2KO cells rarely spread out on Col-1 surface for the first 6 h, and only reached to 9.8% and 27.6% at 24 h in two K2KO Crispr-Cas9 cell lines (#3 and #10). Together, these results demonstrate that Kindlin-2 is essential for cell cytoskeleton integrity, FA formation, and cell spreading in MLO-Y4 cells. These observations also indicate that loss of Kindlin-2 in osteocytes may affect its cellular mechanical responses through the defects in cytoskeleton and FA of these cells.

To further confirm the relationship between Kindlin-2 and in vivo osteocyte morphology, we stained the actin cytoskeleton with Phalloidin-Rhodamine over tibia sections from both control and cKO mice (Fig. 4n and Supplementary movie 2). As presented in Fig. 4N, the osteocytes from control mice displayed a typical ellipsoid cell shape, extended massive connections with neighboring cells, and all were aligned in parallel to bone marrow. These extensive intracellular connections and parallel orientation allow osteocytes a fast and efficient transition and communications during mechanical experiences^69,70^. However, osteocytes with Kindlin-2 deletion altered their morphology in several ways (Fig. 4n). First, the osteocyte cell body in cKO mice presented less elongated cell body with dense F-actin filaments accumulation in the perinuclear region (white arrows). Second, compared to the osteocytes from control mice, osteocytes in cKO mice exhibited less and shorter dendrites extended from cell body (yellow arrows in Fig. 4n zoom-in images), and their dendrites had dramatic discontinuity between osteocytes (yellow asterisks). These observations are similar to the cytoskeleton defects in K2KO MLO-Y4 cells (Fig. 4b, c). Third, cKO mice had more round-up and miss-oriented osteocytes in tibia sections (red arrows). Collectively, these results suggest that Kindlin-2 deletion in osteocytes influences its cytoskeleton organization and FA formation both in vitro and in vivo.

### Fluid shear stress up-regulates Kindlin-2 and down-regulates Sclerostin and Smad2/3 expression in a dose-dependent manner in MLO-Y4 cells

**Fig. 5:**
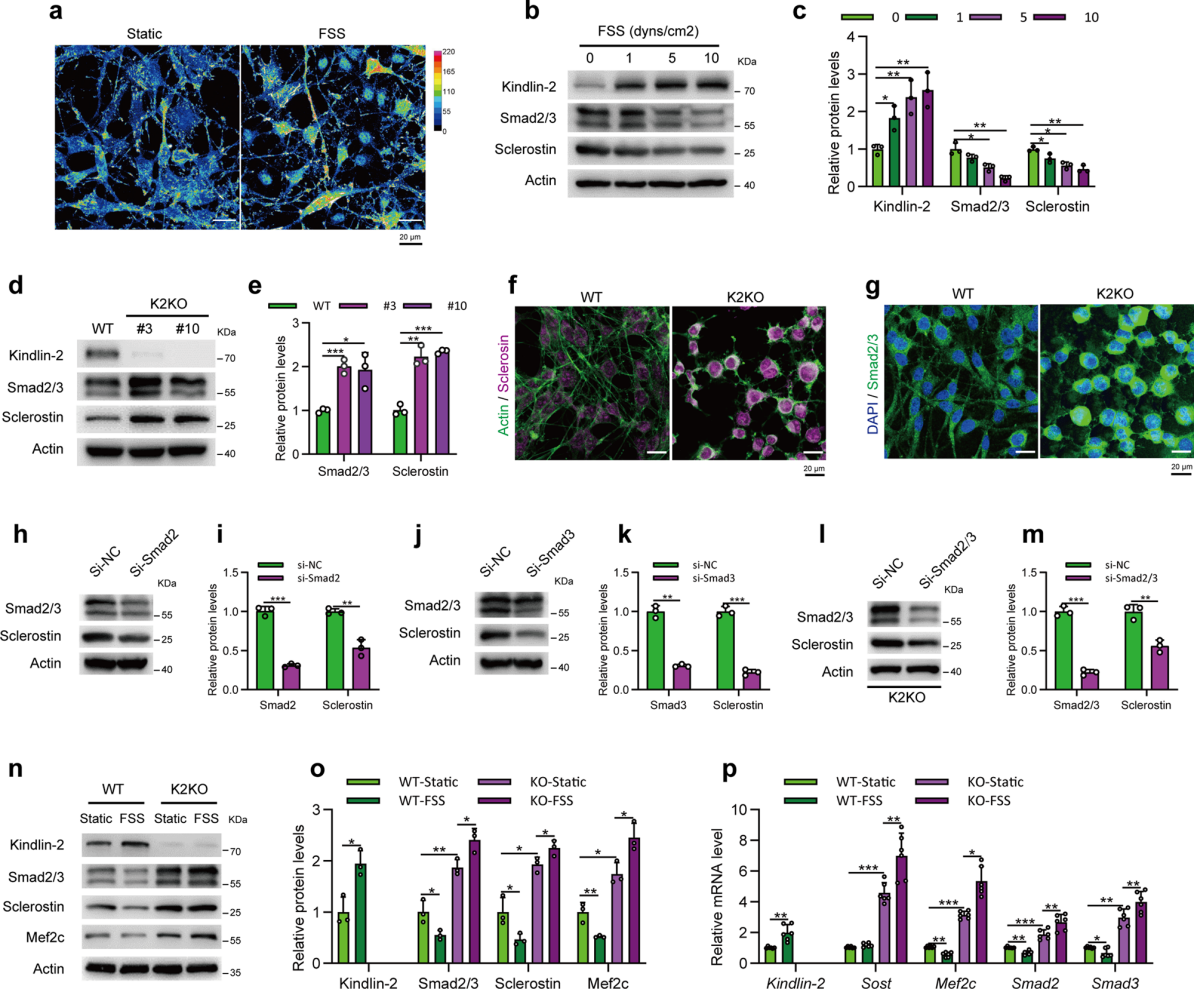
Fluid shear stress (FSS) up-regulates Kindlin-2 and down-regulates Sclerostin and Smad2/3 expression in a dose-dependent manner in MLO-Y4 cells. **a** Heat-map images of Kindlin-2 expression in WT MLO-Y4 cells at static or FSS conditions. **b** Protein levels of Kindlin-2, Smad2/3, Sclerostin, and β-Actin detected with western blotting in WT MLO-Y4 cells under 0, 1, 5, and 10 dyns/cm^2^ FSS treatment. **c** Quantification of protein levels of Kindlin-2, Smad2/3 and Sclerostin relative to that of β-Actin (as a loading control) in **b**. **d** The protein expression of Kindlin-2, Smad2/3 and Sclerostin was analyzed by western blotting in WT and two K2KO (#3, #10) MLO-Y4 cells. β-Actin served as a loading control. **e** Protein levels of Kindlin-2, Smad2/3 and Sclerostin relative to that of β-Actin in WT and K2KO cells were quantified by densitometric analysis of western blotting. **f** Merged immunofluorescence images of Sclerostin (magenta) and F-actin (green) in WT and K2KO MLO-Y4 cells. **g** Merged immunofluorescence images of Smad2/3 (green) and DAPI (blue) in WT and K2KO MLO-Y4 cells. WT MLO-Y4 cells were transfected with control (si-NC) or Smad2 siRNA (**h**, **i**), or Smad3 siRNA (**j**, **k**) for 48 hours and then analyzed by western blotting with antibodies against Smad2/3, Sclerostin and β-Actin (as a loading control). K2KO MLO-Y4 cells were transfected with control (si-NC) or double Smad2/3 siRNA (si-Smad2/3) for 48 h and then analyzed by western blotting (**l**, **m**) with antibodies against Smad2/3, Sclerostin and β-Actin (as a loading control). **n** Protein levels of Kindlin-2, Smad2/3, Sclerostin, Mef2c, and β-Actin detected with western blotting in WT and K2KO MLO-Y4 cells under static or 10 dyns/cm^2^ loading conditions, and quantified in **o**. **p** The transcription levels of *Kindlin-2, Smad2, Smad3, Mef2c,* and *Sclerostin* mRNA were detected through quantitative PCR analysis in WT and K2KO cells under static and FSS conditions. Quantitative results from at least three independent experiments. *P < 0.05, **P < 0.01, ***P < 0.001, versus controls, Student’s *t* test. Results are expressed as mean ± standard deviation (s.d.).

To explore the mechanism behind Kindlin-2 in osteocyte mechanobiology, we first conducted steady fluid shear stress (FSS) treatment in WT MLO-Y4 cells to mimic the mechanical stimulations that osteocytes experience in vivo^71^. As shown in Fig. 5a, we observed an obvious increase of Kindlin-2 expression through immune-fluorescence (IF) staining in MLO-Y4 cells upon FSS treatment. Moreover, western blotting revealed that FSS induced expression of Kindlin-2 protein in a dose-dependent manner in MLO-Y4 cells (Fig. 5b, c). These results further confirm that Kindlin-2 is tightly associated with external mechanical stimulation in osteocytes.

It is known that the expression and secretion of Sclerostin are closely regulated by mechanical stimulation both in vivo^51^ and in vitro^72^. Published study focused on osteoblast cells suggests that traditional TGF-β/Smad signaling is involved in transcription of *Sost* in UMR106.01 mature osteoblast cell line^73^. Therefore, we next tested any possible involvements of Sclerostin and Smad2/3 in FSS through western blotting. Consistent with previous results, we found that FSS stimulation decreased Sclerostin expression in MLO-Y4 cells (Fig. 5b, c). Interestingly, we also detected an obvious reduction of Smad2/3 expression upon FSS treatment (Fig. 5b, c).

### Kindlin-2 suppresses the expression of Sclerostin through Smad2/3 in MLO-Y4 cells

We next analyzed the protein expression of Smad2/3 and Sclerostin in two K2KO Crispr-Cas9 cell lines (#3 and #10) through western blotting (Fig. 5d). In K2KO cells, we observed dramatic increases in expression of both Sclerostin and Smad2/3 proteins relative to that in WT cells (Fig. 5e). Similar increases in the expression of Smad2/3 and Sclerostin were observed in the K2KO cells through IF staining (Fig. 5f, g). These data suggest that Kindlin-2 may function as a suppressor of Smad2/3 and Sclerostin expression in MLO-Y4 cells. A previous study by Loots and coworkers has shown that Smad2/3 transcriptionally activates the expression of Sclerostin in osteoblasts^73^. We found that siRNA knock down of Smad2, Smad3, or both (Smad2/3) dramatically reduced the level of Sclerostin protein in MLO-Y4 cells (Fig. 5h–m). Together, these results suggest that Kindlin-2 suppresses Sclerostin expression in MLO-Y4 cells probably through its suppression of Smad2/3.

To further analyze the influence of Kindlin-2 on Smad2/3 and Sclerostin upon FSS stimulation, we performed FSS at 10 dyns/cm^2^ for 2 h with WT and K2KO cells. As shown in Fig. 5n, under FSS administration, WT MLO-Y4 cells experienced a clear Kindlin-2 upregulation, which was associated with reduced expression of both Smad2/3 and Sclerostin in these cells. However, in the absence of Kindlin-2, the FSS stimulation failed to downregulate neither Smad2/3 nor Sclerostin expression in MLO-Y4 cells (Fig. 5n). Moreover, quantitative analysis of western blot results (Fig. 5o) and qPCR results (Fig. 5p) further confirmed that Kindlin-2 could downregulate Smad2/3 and Sclerostin expression transcriptionally and translationally upon FSS treatment. Published results suggest that Smad2/3 indirectly activates *Sost* transcription through Mef2C, which binds to a distal gene enhancer of the *Sost* promoter, ECR5^73,74^. In MLO-Y4 cells, we found that Mef2C mRNA and protein levels were decreased upon FSS treatment in WT cells (Fig. 5o, p). However, this FSS-induced suppression was abolished in K2KO cells (Fig. 5o, p). Together, these data indicate that FSS inhibits Sclerostin expression at least partially through Kindlin-2-Smad2/3-Mef2C axis in MLO-Y4 cells.

### In vivo loading increases serum Sclerostin, and enhances Sclerostin and Smad2/3 expression in osteocytes of cKO mice

**Fig. 6:**
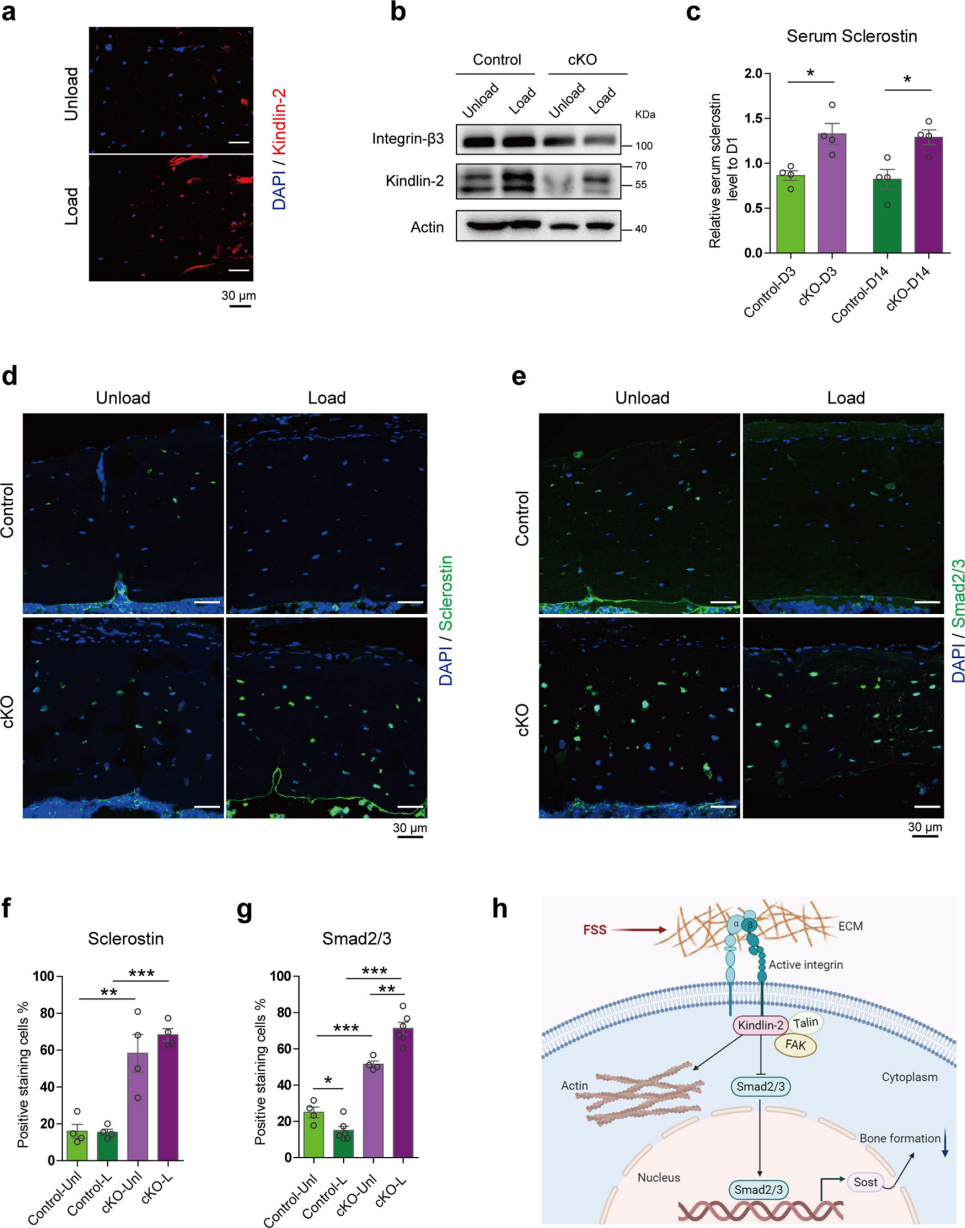
Kindlin-2 deletion in osteocytes enhances Sclerostin and Smad2/3 expression upon mechanical loading in mice. **a** Immunofluorescence staining of Kindlin-2 in the midshaft of tibial cross sections from unloaded and loaded limbs of 4-month-old control male mice. **b** Protein levels of integrin-β3, Kindlin-2, and β-Actin detected with western blotting in 4-month-old control and cKO male mice in unloaded and loaded tibia samples. **c** Serum Sclerostin levels from ELISA detection at loading Day 3 (D3) and Day 14 (D14) relative to Day 1 (D1, before loading) in control and cKO mice. *N* = 4 for each group. **d** IF staining of Sclerostin (green) merged with DAPI (blue) in tibia sections from control and cKO mice. **e** IF staining of Smad2/3 (green) merged with DAPI (blue) in tibia sections from control and cKO mice. Quantitative analysis for Sclerostin-positive (**f**) and Smad2/3-positive (**g**) cells in tibia sections from control and cKO mice. *N* = 4–6 for each group. **h** Working model. *P < 0.05, **P < 0.01, ***P < 0.001, versus controls, Student’s *t* test. Results are expressed as mean ± standard deviation (s.d.).

We further examined expression of Kindlin-2 in osteocyte upon mechanical loading in mice. We conducted fluorescence staining of the unloaded and loaded tibial sections from control mice using anti-Kindlin-2 antibody. Results displayed a significant increase in Kindlin-2 protein expression in osteocytes embedded in mineralizing matrix in the loaded tibial bone (Fig. 6a). Furthermore, western blotting using protein extracts from osteocyte-enriched mid-diaphyseal tibial shafts (with their bone marrow flushed) from age- and sex-matched control and cKO mice revealed that mechanical loading dramatically increased the level of Kindlin-2, which was largely reduced in cKO mice (Fig. 6b).

We next measured the Sclerostin level in the serum of in vivo loading mice. To monitor the serum Sclerostin changes at different loading time points, we collected the serum at Day 3 and Day 14 after in vivo tibia loading and compared it with serum collected at Day 1 (before loading experiments; Fig. 6c). As expected, we observed a clear serum Sclerostin drop in control mice after loading with only 86.3% serum Sclerostin remain at Day 3 and 82.2% at Day 14. However, the serum Sclerostin level in cKO mice was increased after mechanical loading, which showed 32.8% increase at Day 3 and 28.9% increase at Day 14.

With these observations, we next determined the Sclerostin and Smad2/3 expression levels in osteocytes by IF staining over unload and load tibia samples. As presented in Fig. 6d, while mechanical loading did not alter Sclerostin-positive osteocytes in control mice, Sclerostin-positive osteocytes, and the overall Sclerostin expression in each osteocyte were largely increased in cKO mice than those in control mice, especially after mechanical loading (Fig. 6f). Consistent with in vitro data, Smad2/3 expression shared similar patterns, i.e., osteocytes of load tibia had reduced Smad2/3 expression compared with those of unload tibia in control mice, but cKO tibia presented dramatic increase of Smad2/3-positive osteocytes after loading (Fig. 6e, g). Together, these data confirmed that mice with conditional Kindlin-2 deletion in osteocytes experienced Sclerostin and Sma2/3 upregulation upon mechanical stimulation.

Based on these results, our working model proposes an important function of Kindlin-2 in osteocyte mechanobiology (Fig. 6h). Mechanical forces activate the transmembrane integrins through its influence over ECM. The cytoplasmic tails of integrins recruit and activate Kindlin-2 and other FA-associated proteins, such as Talin and FAK. Mechanical forces upregulate Kindlin-2 expression in osteocytes. Kindlin-2 inhibits the expression of *Sost* by, at least in part, downregulation of Smad2/3 in osteocytes under mechanical force-stimulated conditions. Kindlin-2 also influences the organization of actin cytoskeleton.

## Discussion

In the present study, we demonstrate the importance of the focal adhesion protein, Kindlin-2, in the regulation of force adaptation during osteocyte mechanobiology. Through μCT scanning, we find that mice with Kindlin-2 loss in osteocytes exhibit remarkable osteopenia phenotype, which is only restricted to load-bearing bones, such as ulna, tibia, femur, and lumbar spine^48^, but not in calvariae. Considering that the *Dmp1* promoter expression is detected in both calvariae and long bones^75^ and a comparable deletion efficiency of Kindlin-2 in both long bones and calvariae is observed in this study, it is unlikely that the osteopenia in load-bearing bones of cKO mice is due to a result of differential Kindlin-2 deletion between long bones and calvariae.

One explanation for this osteopenia phenotype discrepancy between long bones and calvariae is the gene expressing profiling difference originated from intramembrane ossification and endochondral ossification processes. Our group previously showed that conditional deletion of Kindlin-2 in Prx1-expressing mesenchymal progenitors resulted in neonatal lethality, long bone shortening, and loss of skull vault in mice^41^. This result demonstrates that Kindlin-2 is essential for both intramembrane and endochondral ossification during bone development. Considering the different origins and gene profiling of intramembrane and endochondral osteocytes, it is possible that Kindlin-2 functions differentially in two types of osteocytes, for instance, differential functions in mechanotransduction. However, our μCT scanning results with alveolar bone, in which osteocytes are derived from intramembrane process but under frequent mechanical loading, show a remarkable bone loss in alveolar process from cKO mice, but not in control mice. At the tissue level, strain and load estimation from daily activities showed that fibula and tibia exhibited higher load than calvaria^61,76^. At the cellular level, osteocytes derived from long bones have various gene expression profiles^77^, different cell morphology^60^, and diverse protein secretion upon strain application^76^, compared to osteocytes derived from calvaria. These results suggest that osteocytes in load-bearing bones experience larger and more dynamic mechanical stimulations than osteocytes in calvariae. In short, the osteopenia phenotype and reduced bone mechanical properties in long bones are due to a gradual accumulation of defects associated with Kindlin-2 loss during daily mechanical stimulation in these bones.

Published results showed that, besides osteocyte, *Dmp1-Cre* expression is observed in osteoblasts and stromal cells^78,79^. Therefore, in the current study, we cannot rule out the possible contributions of Kindlin-2 loss in these two cell types to the impairment of the mechanotransduction in cKO bone. However, considering the large number of osteocytes in bone tissue (osteocyte occupying 90–95% of total bone cells, almost 10 times of cell number of osteoblasts^80^) and the contribution of mechanosensation of osteocyte in mechanical stimulation^14^, we believe that this load-bearing bone-related phenotype in cKO mice is primarily due to Kindlin-2 loss in osteocytes.

In this study, we conducted two in vivo mechanical loading models and one in vivo unloading-reloading model for cKO mice and their control littermates. Consistent with previous reports^51,52^, we find that mechanical loading preserves bone mass and stimulates bone formation in control mice. However, Kindlin-2 loss in osteocytes reverses the mechanical loading induced bone formation process, rather decreases the bone mass quantity as well as bone quality in loading limbs. Moreover, mechanical unloading leads to similar bone loss in control and cKO mice. Surprisingly, extending HLU experiment with recovery stage shows that cKO mice fail to restore lost bone mass after recovery. This result indicates that Kindlin-2 deletion in osteocytes cannot reverse, but exacerbate mechanical unloading-induced bone loss. These data also suggest that the molecular mechanism for unloading sensation is different from that in loading sensation. Together, current data demonstrate that Kindlin-2 in osteocytes is mainly involved in mechanical loading sensation process, but with limited contribution in unloading sensation.

Mechanistically, we propose Kindlin-2 as a Sclerostin suppressor that contributes to force-induced bone formation during osteocyte mechanobiology. Here, we demonstrate that Kindlin-2 is an essential negative regulator for Sclerostin expression in mechanical loading process. In cultured MLO-Y4 cells, we show that mechanical stimulation enhances Kindlin-2 expression, which further suppresses expression of both Sclerostin and Smad2/3 under shear stress conditions. Moreover, the expression of Sclerostin is tightly controlled by Smad2/3 proteins in MLO-Y4 cells. Consistent with these in vitro data, enhanced Sclerostin secretion in the serum samples in cKO mice and upregulation of Sclerostin and Smad2/3 in loaded tibia samples from cKO mice were detected. These data together demonstrate that osteocytes with Kindlin-2 deletion fail to suppress Sclerostin expression when stimulated with mechanical loading, resulting in reduced bone formation.

The importance for Kindlin-2 in osteocyte mechanotransduction could be related to its functions in FA. Previous studies showed that FA proteins are essential for bone mass maintenance in mechanical stimulation, such as integrin-β1^81–83^, FAK^84^, and Pinch1/2^85,86^. Our results demonstrate that Kindlin-2 controls osteocyte FA formation, cell spreading, cell attachment, and cell morphology both in vitro and in vivo. Consistent with that Kindlin-2 is an essential scaffold protein in FA^26,33,87^, deletion of its expression reduces integrin β1, integrin β3, Talin1 and FAK expression, and affects the plasma membrane continuousness and cytoskeleton integrity in MLO-Y4 cells. Previous studies demonstrate that FA and cell cytoskeleton are active mechanosensors in osteocytes^17,83^. Our results suggest that the abnormal osteocyte responses to mechanical loading in cKO mice could be resulted from the defects of FA formation and cytoskeleton integrity associated with Kindlin-2 loss in osteocytes.

It should be noted that, in addition to FAs, osteocytes contain several other mechanosensors, including primary cilium, gap junctions, ion channels, cell cytoskeleton, and ECMs^17^. It would be interesting to study the possible involvement of Kindlin-2 in other mechanosensors in future studies.

In short, we identify Kindlin-2 as a novel and important regulator in osteocyte mechanotransduction during the process of force-induced bone formation. The results presented in current study shed new light on the molecular mechanism between mechanical stimulation and Sclerostin suppression in osteocytes.

## Methods

### Animal study

Floxed *Kindlin-2* mice (*Kindlin-2*^*fl/fl*^) were generated as we previously described in Wu et al.^41^. Mice with 10-kb mouse *Dmp1* gene promoter-driving Cre recombinase expression (*Dmp1-Cre*) were generated as described in Lu et al.^58^. We obtained heterozygous mice (*Dmp1-Cre* mice; *Kindlin-2*^*fl/+*^) by crossing *Kindlin-2*^*fl/lf*^ mice with *Dmp1-Cre* mice. By crossing heterozygous mice with *Kindlin-2*^*fl/fl*^ mice, we obtained homozygous mice (*Dmp1-Cre* mice; *Kindlin-2*^*fl/fl*^). Next, we crossed male *Dmp1-Cre* ; *Kindlin-2*^*fl/fl*^ mice with female *Kindlin-2*^*fl/fl*^ mice. As a result, we obtained conditional knockout *Dmp1-Cre* mice; *Kindlin-2*^*fl/fl*^ mice (cKO); and their control littermates (e.g., *Kindlin-2*^*fl/fl*^). All research protocols were approved by the Institutional Animal Care and Use Committee (IACUC) of Southern University of Science and Technology.

### Micro-computerized tomography (μCT)

For in vivo μCT, mice were anesthetized with 2.5% Avertin (100 μl per 10 g body weight) and subjected for μCT scan. For ex vivo micro μCT, calvaria and long bones (femur, tibia, and ulna) were dissected and immediately fixed with 4% PFA for 24 h and post fixed with 70% ethanol at 4 °C. Either live or fixed non-demineralized bones were subjected to μCT analyses in the Department of Biology of Southern University of Science and Technology using a Bruker μCT (SkyScan 1172 Micro-CT, Bruker Micro-CT, Kontich, Belgium). The scan resolution was 13 μm for tibiae and calvariae, 16 μm for ulna and femur with setting of 60 kV, 100 μA and AI 0.5 mm filter. A lower/upper threshold of 80/255 and 60/255 were used to segment bone from other tissues in ex vivo and in vivo μCT scan, respectively. Calvaria parameters were analyzed in ROI at the center of skull with 2 mm×2 mm size. Ulna parameters were assessed at the proximal spongiosa from the end of growth plate to 1200 μm extending distally for trabecular bone, and 500 μm thick of cortical bone at the midshaft for both ulna and radius cortical bones. Tibia parameters were assessed as described in Sugiyama et al.^88^, i.e., at the proximal spongiosa from the end of growth plate to 750 μm extending distally for trabecular bone, and from the 37% position of tibia length extending to 500 μm distally for cortical bone. Femur parameters were assessed from the distal growth plate; the trabecular bone rages from 500 μm to 2000 μm; the cortical bone rages from 4000 μm to 5000 μm. The parameters for cortical bone included the bone mineral density (BMD, g/cm^3^), bone volume fraction (BV/TV), and cortical thickness (Ct.Th, mm). The parameters for trabecular bone included the bone mineral density (BMD, g/cm^3^), bone volume fraction (BV/TV), trabecular thickness (Tb.Th, μm), trabecular separation (Tb.Sp, μm), and trabecular number (Tb.N, 1/mm).

### In vivo ulna loading and tibia loading

Force loading experiments were conducted as previously described in refs. ^89,90^ using an electro actuator (Bose ElectroFore 3200; EndureTEC Minnetonka, MN, USA). Briefly, mice were under anesthesia with 2.5% Avertin injection (100 μl per 10 g body weight). For ulna loading, the right ulna was loaded with cyclic compression force (2 Hz, sine waveform, 2.5 N peak fore, 450 cycles per loading). For tibia loading, the right tibia was loaded with cyclic compression force (4 Hz, triangular waveform, 9.0 N peak force, 1200 cycles per loading). The left limbs were intact and served as unloading internal controls. Mice were loaded on alternative day for 2 weeks. Injection of calcein (30 mg/kg body weight; Sigma, Saint Louis, MO, USA) was administered at Day 4 and Day 12, respectively. Animals were killed on Day 14 for bone histomorphometry.

### In vivo hindlimb unloading

Hindlimb unloading experiments were conducted as described before in Robling et al.^51^. Specifically, 14-week-old male mice were outfitted with tail harnesses and their hindlimbs suspended in air and lose the ground reaction forces. HLU experiments were designed for 21 days. The HLU-recovery experiments included 21 days HLU treatment followed by another 21 days free exploration stage. The experimental control mice were husbandry with normal activities.

### Femur three-point bending

Femurs were dissected free of soft tissue and immediately kept in 1x PBS at 4 °C. Samples were kept in wet and tested right after dissection or 1 day after dissection. The strength test was performed at the midshaft of femur. The machine used for this test is ElectroForce (Bose ElectroFore 3200; EndureTEC Minnetonka, MN, USA) with continuous displacement of 0.05 mm/sec in a single stop setting (Ramp waveform, span length, 7 mm). Whole femur mechanical properties, including maximum load, maximum displacement, and stiffness, were determined using load-displacement diagrams.

### Bone histology and immunohistochemistry

For double calcein labeling samples, the bones were embedded in methyl methacrylate following the manufactory plastic embedding protocol (EM0200, Osteo-Bed Bone Embedding Kit, Sigma, MO, USA). Transverse section in 6 μm thickness of tibiae and ulnas were obtained by cutting with an annular diamond saw. Images of double calcein labeling bone sections were visualized using the argon 488 nm laser of fluorescent microscopy (Olympus, BX53). For bone immunohistochemistry (IHC) and immunofluorescence (IF), bone samples were decalcified with 10% ethylenedinitrilotetraacetic acid (EDTA, Sigma) for 2 weeks and 3 weeks for ulna and tibia samples, respectively. Samples were embedded in paraffin and cut in 5 μm transversal sections. IHC and IF experiments were performed using our standard protocols as we previously descripted^41,48^. Antibodies used in this study are listed in Supplementary Table 3.

### Serum ELISA

Serum samples were collected from the supernatant of centrifuged (4°C, 12,000×*g*, 20 min) mouse whole blood after coagulation for 1 h at room temperature. Samples were immediately kept at -80°C fridge before usage. Serum levels of Sclerostin were measured by a Mouse/Rat Sost Immunoassay (R&D systems, Inc., Minneapolis, MN, USA, cat#: MSST00).

### Immunofluorescence and confocal analysis

Cells were cultured on glass coverslips or rat tail Collagen-I coated coverslips (Corning, cat# 354236, 40 ng/cm^2^) for 24–48 h. Cultured cells were fixed with 4% PFA, penetrated with 0.25% Triton X-100, blocked with 1% BSA and then incubated with antibodies. Antibodies used in this study are listed in Supplementary Table 3. Live imaging and Z-stack imaging were conducted by Nikon A1R laser-scanning confocal microscopy with 40x objective set at 5 s time interval and 0.5 μm z-stack interval.

### Cell culture and transfection

Kindlin-2 deletion in MLO-Y4 cells was generated as previously described in^48^. Cells were maintained in α-MEM with 10% FBS and 1% P/S, in 37 °C, 5% CO_2_ cell culture incubator. For transient transfection, 60–80% confluent cells were transfected with indicated siRNA using the Lipofectamine ® RNAiMAX Reagent according to the manufacturer’s instructions. SiRNA target sequences were listed in Supplementary Table 2.

### Quantitative real-time PCR and western blot analyses

RNA and protein isolation, quantitative real-time PCR, and western blot analyses were performed as previously described^91^. In brief, total RNA was extracted from cultured cells using Trizol reagents. Synthesis of cDNA was performed using 2 μg of RNA by a Transcriptor First Strand cDNA Synthesis Kit according to the manufacturer’s instructions. Relative mRNA expression levels were determined using a SYBR Green qPCR kit with CFX96 Real-Time System. β-Actin mRNA was used for normalization. The specific primers for gene expression analysis were listed in Supplementary Table 1. For the protein samples extracted from bones, calvariae were dissected from control and cKO mice free of muscle or other tissues; long bone samples were dissected free of other tissues, flashed out the bone marrow and only kept the osteocyte-enriched mid-diaphyseal shafts. For western blotting, cell lysates were harvested in RIPA lysis buffer. Protein concentration was measured with a BCA kit. Aliquots of 30 μg total protein were separated and transferred onto a PVDF membranes. Membranes were blocked at room temperature in 5% non-fat powdered milk for 1 h, followed by an overnight incubation at 4 °C with primary antibodies. The specific primary antibodies for western blotting were listed in Supplemental Table 3. After incubation with appropriate HRP-conjugated secondary antibodies, blots were developed using an enhanced chemiluminescence and exposed in ChemiDoc XRS chemiluminescence imaging system.

### Fluid-induced flow shear stress treatment

Fluid-induced flow shear stress was performed with Streamer ® System STR-4000 (Flexcell International Corporation, Burlington, NC, USA) as discussed previously in Chen et al.^92^. MLO-Y4 cells with a total cell number of 3.0 × 10^5^ cells were seed on Collagen-I pre-coated culture slips (75 mm × 25 mm × 1 mm, Flexcell) for 48 h before FSS treatment. Upon FSS treatment, the culture slips were transferred into a parallel plat flow chamber and cells were exposed to 1, 5, or 10 dyne/cm^2^ fluid flow for 2 h. For static controls, MLO-Y4 cells were kept in incubator without any further treatment. Protein and mRNA samples were collected right after FSS treatment.

### Scanning electron imaging

Wild-type and Kindlin-2 knockout MLO-Y4 cells were seed on 12-mm glass coverslips at a density of 1.0 × 10^4^ cells/slip and cultured for 24 h. Cells were fixed with cold 100% methanol at −20 °C for 15 min. SEM sample preparation was conducted as previously descripted^93^. Cells were dehydrated by incubation in a series of methanol solutions for 10 min per solution: 35%, 50%, 75%, 90%, and 100% methanol. Cells were completely dehydrated with hexamethyldisilazane (HMDS) treatment for 10 min and left to air dry overnight. The samples were mounted using double-sided conductive tapes, and coated with Au/Pt (Gold/Platinum) particles in pumper to increase the sample conductivity. The cellular morphology of MLO-Y4 cells was observed on scanning electron microscope (SEM, ZEISS Merlin) at 5.0 kV. For permeabilization, cells were incubated with 0.15% Triton X-100 in 1x PBS for 60 s before fixation^66^.

### Nano-indentation

The indentation experiments were performed with a Nano Indenter G200 (Keysight Technologies, Inc., Santa Rosa, CA, USA), equipped with a three-sided pyramid Berkovich diamond tip. The identical loading scheme applied consists of a loading stage at a constant rate of 20 mN/min to a depth of 1000 nm, holding at this load for a period of 10 s and then unloading to 15% of the peak load at a rate of 10 mN/min. Each sample was performed on twenty indentations at the midshaft of bone samples. For the indenter tip, Young’s modulus (Ei) = 1140 GPa and Poisson’s ratio (vi) = 0.07. In calculating the modulus values from the nano-indentation data, the Poisson ratio for bone is taken to be 0.3, as suggested in previous literature^94^.

### Statistics and reproducibility

All data were analyzed in this study by using the GraphPad Prism software (Version 5.0). The differences between two groups were analyzed by two-tailed Student’s *t* test. The differences among different time points were analyzed by two-way ANOVA. Results are expressed as mean ± standard deviation (s.d.). Difference with *P* < 0.05 was considered as statistically significant. All experiments were repeated at least three times. Highly reproducible results were obtained.

### Reporting summary

Further information on research design is available in the Nature Research Reporting Summary linked to this article.

## Supplementary information

Supplementary Information

Description of Additional Supplementary Files

Supplementary Movie 1

Supplementary Movie 2

Supplementary Data 1

Reporting Summary

## Data Availability

All data generated or analyzed during this study are included in the Supplementary Data 1. Any other data are available from the corresponding author upon reasonable request.
