# Supplementary material for: Kindlin-2 mediates mechanotransduction in bone by regulating expression of Sclerostin in osteocytes
Source: Commun Biol. 2021 Mar 25;4:402. doi: 10.1038/s42003-021-01950-4 (PMC7994671; doi:10.1038/s42003-021-01950-4)
Supplement: Supplementary file 1 — Supplementary Information [file 42003_2021_1950_MOESM1_ESM.pdf]

Supplementary information

Supplementary figures

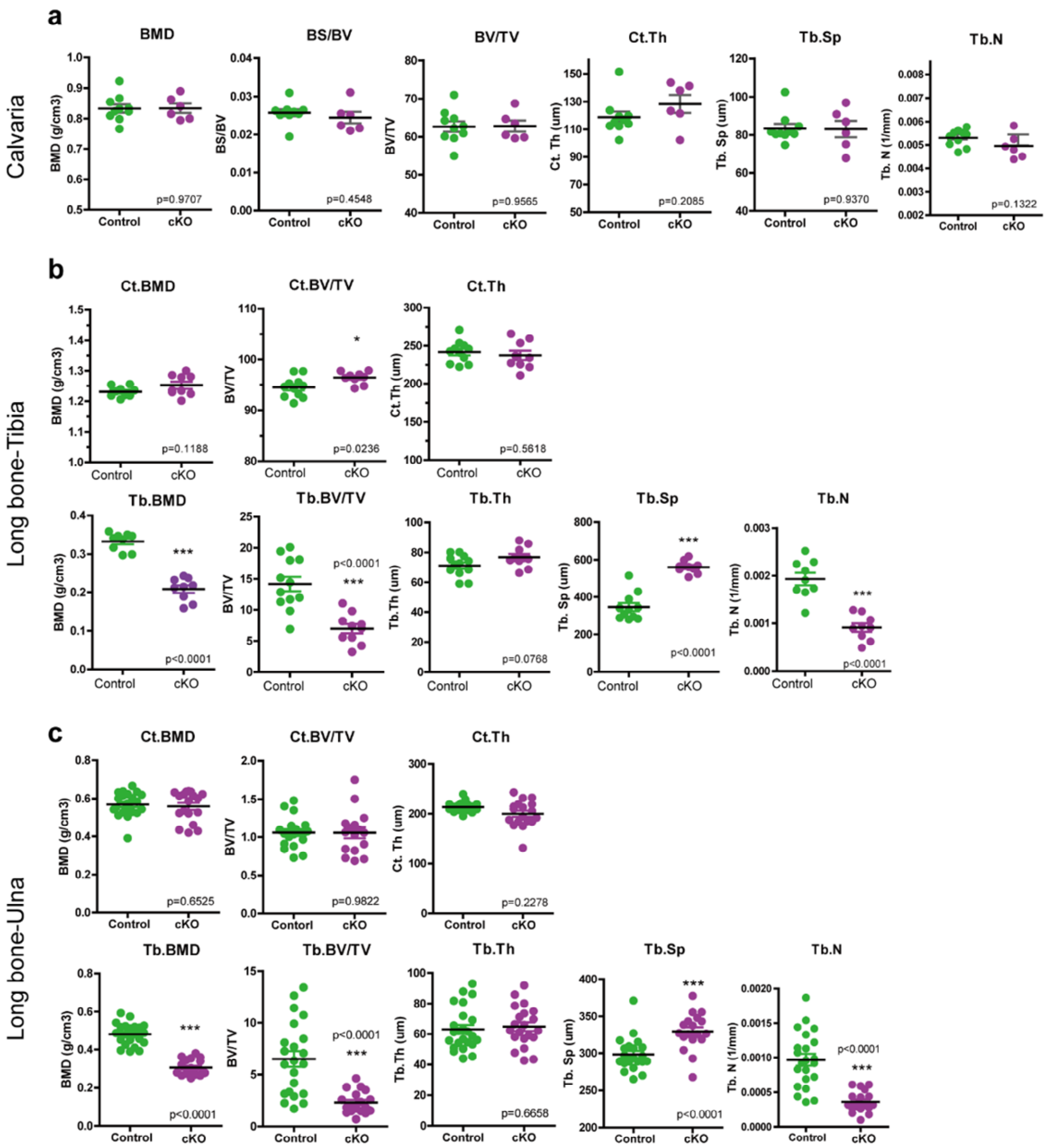

Supplementary Figure. 1. µCT scanning results of calvaria and long bones from control and cKO mice. (**a**) Statistical analyses of bone mineral density (BMD), bone volume fraction (BS/BV), bone volume/tissue volume (BV/TV), cortical thickness (Ct.Th), trabecular separation (Tb.Sp), and trabecular number (Tb.N) of µCT scanning ROI of 2x2 mm calvaria from

control and cKO mice. N = 6~10 for each group. (**b**) Quantitative analyses of BMD, BV/TV, Ct.Th, Tb.Th, Tb.Sp, and Tb.N of midshaft (cortical) bone and proximal spongiosa (trabecular bone) from control and cKO mice. N = 10~12 for each group. (**c**) Statistical analysis of μCT scanning over the midshaft (cortical) and proximal spongiosa (trabecular) of ulna for control and cKO mice. N = 18~22 for each group. **P* < 0.05, ***P* < 0.01, ****P* < 0.001, versus controls, Student's *t* test. Results are expressed as mean ± standard deviation (s.d.).

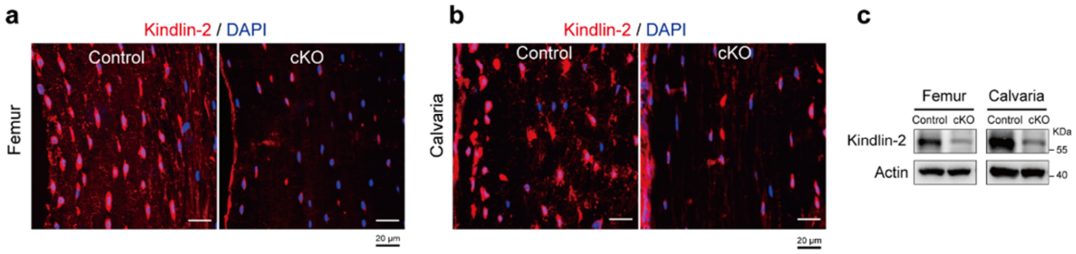

Supplementary Figure. 2. Kindlin-2 expression in long bones and calvariae. Immuno-fluorescence staining of Kindlin-2 in the cross-section of distal femur bones (**a**) and calvariae (**b**) from 5-month-old control and cKO male mice. Kindlin-2 in red, DAPI in blue. (**c**) Western-blot detection of Kindlin-2 in bone samples from 5-month-old control and cKO male mice. These four lanes were from the same blot with the same exposure time.

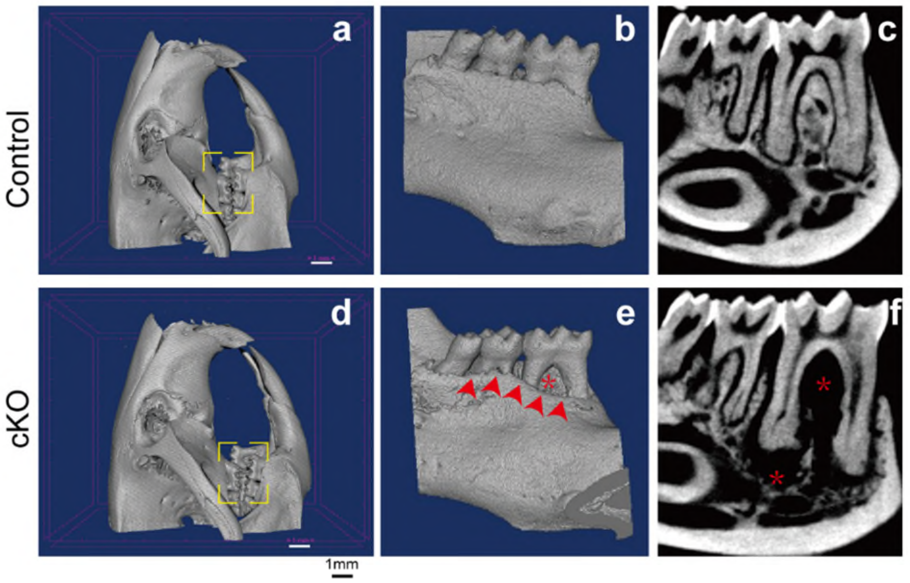

Supplementary Figure. 3. μCT scanning results of alveolar bone from both control and cKO mice. (**a**, **d**) 3D reconstruction image of overall head for 7-month-old control and cKO male mice. Yellow squares highlight the location of alveolar bones. (**b**, **e**) 3D reconstruction image of alveolar bones for control and cKO mice. Red asterisk indicates the significant bone loss in cKO mice; Red arrow-heads point to progress tooth root exposure in cKO mice. (**c**, **f**) Cross-section of alveolar bones for control and cKO mice. Red asterisks refer to the trabecular bone loss in cKO mice.

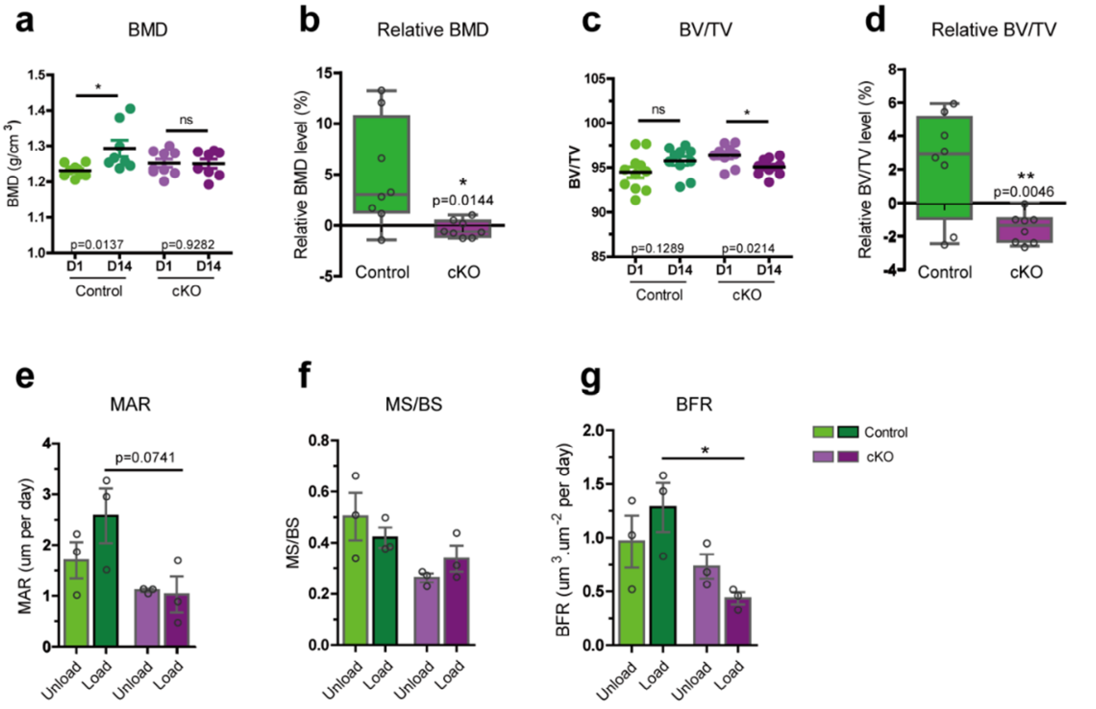

Supplementary Figure 4. Mice lacking Kindlin-2 in osteocytes exhibit defective skeletal responses to external mechanical tibia loading. (**a-d**) Quantification analysis of BMD and BV/TV for right tibia cortical bone before (D1) and after (D14) mechanical loading stimulation in control and cKO mice. N = 8~9 for each group. (**e-g**) Quantification of MAR, MS/BS and BFR in unload (left) tibia and load (right) tibia in control and cKO mice. N = 4 for each group. *$P < 0.05$, **$P < 0.01$, ***$P < 0.001$, versus controls, Student's t test. Results are expressed as mean ± standard deviation (s.d.).

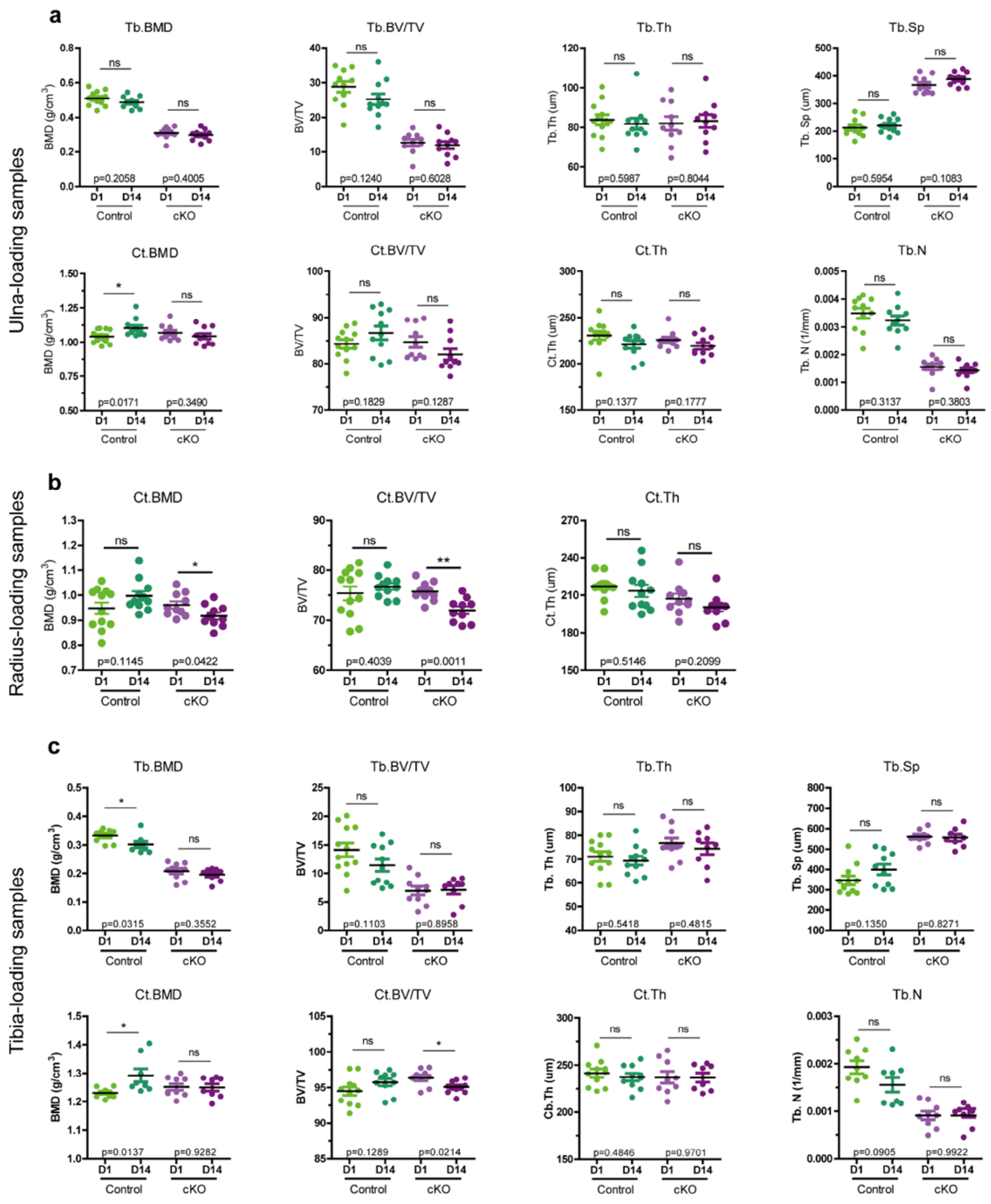

Supplementary Figure 5. μCT scanning results of right loading limbs before (Day 1) and after (Day 14) mechanical simulation in control and cKO mice. (**a**) Ulna-loading samples. N = 10~12 for each group. (**b**) Radius-loading samples. N = 10~12 for each group. (**c**) Tibia-loading samples. N = 8~9 for each group.

Supplementary Tables

Supplementary Table 1: Mouse qPCR primers.

Name	5' primer	3' primer
Actin	GGCTGTATTCCCCTCCATCG	CCAGTTGGTAACAATGCCATGT
Kindlin-2	TGACGGGATAAGGATGCCA	TGACATCGAGTTTTTCCACCAAC
Smad2	ATGTCGTCCATCTTGCCATTC	AACCGTCCTGTTTTCTTTAGCTT
Smad3	CACGCAGAACGTGAACACC	GGCAGTAGATAACGTGAGGGA
Mef2c	ATCCCGATGCAGACGATTCAG	AACAGCACACAATCTTTGCCT
Sost	AGCCTTCAGGAATGATGCCAC	CTTTGGCGTCATAGGGATGGT

Supplementary Table 2: SiRNA target sequences.

Name	5' primer	3' primer
SiNC	UUCUCCGAACGUGUCACGUTT	ACGUGACACGUUCGGAGAATT
SiSmad2	GAAAUGACAAGAAGACAUATT	UAUGUCUUCUUGUCAUUUCTT
SiSmad3	GCGGAGUGCCUCAGUGACATT	UGUCACUGAGGCACUCCGCTT

Supplementary Table 3: Antibodies information.

Name	Supplier	Category number
Connexin-43	Abcam	Ab11370
FAK	Abcam	Ab40794
p-FAK (Tyr397)	Abcam	Ab81298
Kindlin-2	Protein Tech	11453-1-AP
Kindlin-2	Sigma-Aldrich	mAB2617
Integrin β1	Cell Signaling	#34971
Integrin β3	Cell Signaling	#13166
Smad2/3	Cell Signaling	#8685
p-Smad2-S465/467+p-Smad3-S423/425	ABclonal	AP0548
Sclerostin	Abcam	Ab63097
Mef2c	Cell Signaling	#5030
Talin-1	Cell Signaling	#4021
p-Talin-1 (S425)	Cell Signaling	#13589
