# Supplementary material for: Kindlin-2 mediates mechanotransduction in bone by regulating expression of Sclerostin in osteocytes
Source: Commun Biol. 2021 Mar 25;4:402. doi: 10.1038/s42003-021-01950-4 (PMC7994671; doi:10.1038/s42003-021-01950-4)
Supplement: Supplementary file 2 — Description of Additional Supplementary Files [file 42003_2021_1950_MOESM2_ESM.pdf]

Description of Additional Supplementary Files

File name: Supplementary Movie 1

Description: Live tracking of cell spreading and attachment within the first 30 minutes after seeding in WT and K2KO MLO-Y4 cells.

File name: Supplementary Movie 2

Description: Z-stack tracking for F-actin cytoskeleton in tibia section samples from control and cKO mice.

File name: Supplementary Data 1

Description: Source data for Main and Supplementary Figures.
