# Supplementary material for: Kindlin-2 mediates mechanotransduction in bone by regulating expression of Sclerostin in osteocytes
Source: Commun Biol. 2021 Mar 25;4:402. doi: 10.1038/s42003-021-01950-4 (PMC7994671; doi:10.1038/s42003-021-01950-4)
Supplement: Supplementary file 6 — Reporting Summary [file 42003_2021_1950_MOESM6_ESM.pdf]

nature research

Corresponding author(s): Guozhi Xiao

Last updated by author(s): Feb 19, 2021

Reporting Summary

Nature Research wishes to improve the reproducibility of the work that we publish. This form provides structure for consistency and transparency in reporting. For further information on Nature Research policies, see our Editorial Policies and the Editorial Policy Checklist.

Statistics

For all statistical analyses, confirm that the following items are present in the figure legend, table legend, main text, or Methods section.

n/a	Confirmed	
☐	☒	The exact sample size (*n*) for each experimental group/condition, given as a discrete number and unit of measurement
☒	☐	A statement on whether measurements were taken from distinct samples or whether the same sample was measured repeatedly
☐	☒	The statistical test(s) used AND whether they are one- or two-sided<br>*Only common tests should be described solely by name; describe more complex techniques in the Methods section.*
☒	☐	A description of all covariates tested
☒	☐	A description of any assumptions or corrections, such as tests of normality and adjustment for multiple comparisons
☐	☒	A full description of the statistical parameters including central tendency (e.g. means) or other basic estimates (e.g. regression coefficient) AND variation (e.g. standard deviation) or associated estimates of uncertainty (e.g. confidence intervals)
☐	☒	For null hypothesis testing, the test statistic (e.g. *F*, *t*, *r*) with confidence intervals, effect sizes, degrees of freedom and *P* value noted<br>*Give P values as exact values whenever suitable.*
☒	☐	For Bayesian analysis, information on the choice of priors and Markov chain Monte Carlo settings
☒	☐	For hierarchical and complex designs, identification of the appropriate level for tests and full reporting of outcomes
☒	☐	Estimates of effect sizes (e.g. Cohen's *d*, Pearson's *r*), indicating how they were calculated

Our web collection on statistics for biologists contains articles on many of the points above.

Software and code

Policy information about availability of computer code

Data collection	No software was used.
Data analysis	No software was used.

For manuscripts utilizing custom algorithms or software that are central to the research but not yet described in published literature, software must be made available to editors and reviewers. We strongly encourage code deposition in a community repository (e.g. GitHub). See the Nature Research guidelines for submitting code & software for further information.

Data

Policy information about availability of data

All manuscripts must include a data availability statement. This statement should provide the following information, where applicable:

- Accession codes, unique identifiers, or web links for publicly available datasets
- A list of figures that have associated raw data
- A description of any restrictions on data availability

The authors declare that the data supporting the findings of this study are available within the paper and its supplementary information files.

Field-specific reporting

Life sciences study design

All studies must disclose on these points even when the disclosure is negative.

Sample size	The sample size used for each experiments were listed in the figure legend. The sample size used in basal phenotype detection, including micro CT scanning and 3-point-bending experiments, ranges from 10 to 20 for each group. The sample size used for in vivo loading experiments ranges from 8 to 12, and unloading experiments was 5 mice. The sample size for MAR, Serum Elisa and immuno-fluorescence staining was 3 for each group.
Data exclusions	For in vivo experiments, 1 or 2 mice were excluded from data set. These excluded samples were unhealthy mice who lost 20% of total body weight during or after experiments.
Replication	For experimental animal, normally 5-8 replicates were used for each genotype. For in vitro culture experiments, FSS, WB, qPCR and immuno-staining were replicated at least for 3 times.
Randomization	Samples were selected randomly. KO and their age-matched Control littermates were used.
Blinding	IF, MAR, BFR and micro-CT analyses were performed in double blinded way.

Reporting for specific materials, systems and methods

We require information from authors about some types of materials, experimental systems and methods used in many studies. Here, indicate whether each material, system or method listed is relevant to your study. If you are not sure if a list item applies to your research, read the appropriate section before selecting a response.

Materials & experimental systems

n/a	Involved in the study
☐	☒ Antibodies
☐	☒ Eukaryotic cell lines
☒	☐ Palaeontology and archaeology
☐	☒ Animals and other organisms
☒	☐ Human research participants
☒	☐ Clinical data
☒	☐ Dual use research of concern

Methods

n/a	Involved in the study
☒	☐ ChIP-seq
☒	☐ Flow cytometry
☒	☐ MRI-based neuroimaging

Antibodies

Antibodies used	Connexin-43 (Abcam, Cat# Ab11370), FAK (Abcam, Ab40794), p-FAK-Tyr397 (Abcam, Ab81298), Kindlin-2 (Protein-Tech, Cat# 11453-1-AP), Kindlin-2 (Sigma-Aldrich, Cat# mAB2617), Integrin-beta1 (Cell Signaling, Cat# 34971), Integrin-beta3 (Cell Signaling, Cal# 13166), Smad2/3 (Cell Signaling, Cat# 8685), p-Smad2-S465/467+p-Smad3-S423/425 (ABclone, Cat# AP0548), Sclerostin (Abcam, Cat# Ab63097), Mef2c (Cell Signaling, Cat# 5030), Talin-1 (Cell signaling, Cat# 4021), p-Talin-1-S425 (Cell signaling, Cat# 13589).
Validation	For the species and sensitivity of primary antibodies, the validation was referred to the manufactury's website.

Eukaryotic cell lines

Policy information about cell lines

Cell line source(s)	MLO-Y4 was kindly provided by Dr. Bai, Xiaochun of the Sourtern Medical University
Authentication	Dr. Bai, Xiaochun
Mycoplasma contamination	MLO-Y4 cell line was tested negative for mycoplasma contamination.
Commonly misidentified lines (See ICLAC register)	NA

Animals and other organisms

Policy information about studies involving animals; ARRIVE guidelines recommended for reporting animal research

Laboratory animals	Mice, C57BL6 strain, male, 4-month to 14-month old
Wild animals	This study did not involve wild animals.

Field-collected samples	This study did not involve samples collected from the field.
Ethics oversight	All research protocols were approved by the Institutional Animal Care and Use Committee (IACUC) of Southern University of Science and Technology.

Note that full information on the approval of the study protocol must also be provided in the manuscript.
